# Induction of antitumor immunity through xenoplacental immunization

**DOI:** 10.1186/1479-5876-4-22

**Published:** 2006-05-25

**Authors:** Zhaohui Zhong, Kornel P Kusznieruk, Igor A Popov, Neil H Riordan, Hamid Izadi, Li Yijian, Salman Sher, Orest M Szczurko, Michael G Agadjanyan, Richard H Tullis, Amir Harandi, Boris N Reznik, Grigor V Mamikonyan, Thomas E Ichim

**Affiliations:** 1 The Second Xiangya Hospital of Central South University, Changsha, China; 2 MedVax Pharma Corp, Toronto, Canada/San Diego, USA; 3 Department of Surgery, University of Western Ontario, London, Canada; 4 Medistem Laboratories Inc, Tempe Arizona, USA; 5 Division of Cardiology, Emory University, Atlanta, USA; 6 Canadian College of Naturopathic Medicine, Toronto, Canada; 7 Institute of Molecular Medicine, Huntington Beach, California, USA; 8 Aethlon Medical, Inc, San Diego, California, USA; 9 Department of Medicine, Columbia University, New York, USA; 10 bioRASI LLC, Miami, USA; 11 OncoMune LLC, Los Angeles, California, USA

## Abstract

Historically cancer vaccines have yielded suboptimal clinical results.  We have developed a novel strategy for eliciting antitumor immunity based upon homology between neoplastic tissue and the developing placenta. Placenta formation shares several key processes with neoplasia, namely: angiogenesis, activation of matrix metalloproteases, and active suppression of immune function. Immune responses against xenoantigens are well known to break self-tolerance. Utilizing xenogeneic placental protein extracts as a vaccine, we have successfully induced anti-tumor immunity against B16 melanoma in C57/BL6 mice, whereas control xenogeneic extracts and B16 tumor extracts where ineffective, or actually promoted tumor growth, respectively. Furthermore, dendritic cells were able to prime tumor immunity when pulsed with the placental xenoantigens. While vaccination-induced tumor regression was abolished in mice depleted of CD4 T cells, both CD4 and CD8 cells were needed to adoptively transfer immunity to naïve mice. Supporting the role of CD8 cells in controlling tumor growth are findings that only freshly isolated CD8 cells from immunized mice were capable of inducing tumor cell caspases-3 activation ex vivo. These data suggest feasibility of using xenogeneic placental preparations as a multivalent vaccine potently targeting not just tumor antigens, but processes that are essential for tumor maintenance of malignant potential.

## Introduction

For successful pregnancy to occur, establishment of hemochorial placentation is essential. The process of placenta formation involves tissue morphogenesis [1], activation of matrix metalloproteases [2], and active suppression of immune function [3,4]. Placental expression of matrix metalloproteases (MMPs), [5,6], Fas ligand (Fas-L) [7] and immune suppressants such as DAF [8], CD55 [9], IL-10 [10], and VEGF [11] are involved in generating a suitable placental microenvironment for healthy pregnancy. The expression of the same molecules by tumor cells is associated with poor prognosis and endows a competitive advantage to neoplastic cells [12-16]. In addition, various tumor antigens such as hCG, MAGE, BAGE are expressed in placental tissue [17,18].

Induction of immune responses towards self-proteins associated with tumor progression is a possible therapeutic approach to cancer. While administration of syngeneic or allogeneic proteins induces poor immune response, it is reported that administration of xenogeneic homologous proteins are capable of eliciting immunity against the endogenous self-protein. Vaccination with xenogeneic FGF-R [19], VEGF [20], PAP [21], and MMP-2 [22] resulted in potent immunological control of tumors that depend on these molecules for survival. Indeed, vaccination with xenogeneic endothelial cells has been used to block tumor angiogenesis in the recipient in an effective manner [23-25].

Based on the above rationale, we hypothesized that immunization with xenogeneic placental extracts would result in a potent immune response to the inhibitory cytokines, MMPs, and immune suppressive factors produced by the cancer. The success of this approach would pave the way for cancer vaccines effective against a wide variety of tumors since it does not involve targeting tissue-specific markers, but molecules essential for the existence and propagation of the tumor. Accordingly, in this study we sought to demonstrate feasibility of such an approach using the B16 murine model of melanoma, an accepted model of immunotherapy. We have demonstrated the ability of a single immunization to inhibit tumor growth in a CD4+ T cell dependent manner and the need for both CD4 and CD8 T cells to adoptively transfer immunity. Furthermore immunization using syngeneic dendritic cells pulsed with xenogenic placental extracts induced immunity at the time of tumor inoculation in a semi-therapeutic model. These data demonstrate feasibility of using this novel antigenic source as a starting point for therapeutic vaccine development.

## Materials and methods

### Animals

Female C57/BL6 mice (The Jackson Laboratories, Bar Harbor, ME), 5 wk of age, were kept in filter-top cages at the Animal Care and Veterinary Services Facility, the University of Western Ontario according to the Canadian Council for Animal Care Guidelines. Mice were fed by food and water ad libitum and allowed to settle for 2 wk before initiation of experimentations

### Preparation of xenogeneic placental extract and controls

Porcine placental tissue was obtained from delivering sows and washed in sterile phosphate buffered saline (PBS) containing 5% penicillin streptomycin mixture and placed on ice for transportation. Vascularized placental tissue was homogenized with a tissue grinder and exposed to 4 freeze-thaw cycles alternating from liquid nitrogen to 42 Celsius water bath. Cell debris was pelleted by centrifugation at 1500 g for 45 minutes. Supernatant was collected and sterilized with 0.2 micron Millipore filters. Total protein concentration was determined using the Bradford Assay (BioRad). For control tissue, porcine liver and B16 melanoma cell line proteins were isolated using identical protocol. Experiments utilizing allogeneic placental extracts were performed using term-BALB/c placental extracts prepared in a manner identical to the xenogeneic placental extracts. The whole protein preparations were dissolved into sterile, injection-grade PBS at a concentration of 2 mg/ml, and injections of 50 uL (total mass 10 ug) were performed subcutaneously into C57/BL6 mice 7 days before tumor challenge.

### Pulsing of dendritic cells

At Day 0, bone marrow cells were flushed from the femurs and tibias of C57/BL6 mice, washed and cultured in 6-well plates (Corning, NY) at 4 × 10^6 ^cells/well in 4 ml of complete medium (RPMI 1640 supplemented with 2 mM L-glutamine, 100 U/ml penicillin, 100 μg of streptomycin, 50 μM 2-ME, and 10% FCS (all from Life Technologies, Ontario, Canada) supplemented with recombinant GM-CSF (10 ng/ml; PeproTech, Rocky Hill, NJ) and recombinant mouse IL-4 (10 ng/ml; PeproTech). All cultures were incubated at 37°C in 5% humidified CO_2_. Non-adherent cells were removed after 48 h of culture (Day 2) and fresh medium was added. After 7 days of culture, >90% of the cells expressed the characteristic DC-specific marker CD11c asdetermined by FACS. DC were washed and plated in 24-well platesat a concentration of 2 × 10^5 ^cells/well in 400 μl ofserum-free RPMI 1640. Pulsing with XPE and/or OVA was performed by addition of said antigens at a concentration of 10 ug/ml for 24 hours.

### Proliferation assays

Proliferative recall responses to XPE and ovalbumin in immunized mice were assessed by sacrificing C57/BL6 mice, 14 days after immunization with antigen-loaded DC. T cells were purified from suspensions of lymph nodes using CD4+ T cell column (R&D Systems) after washing in PBS. Purified T cells were cultured in 96 well plates with irradiated syngeneic splenocytes in triplicate and mixed with serial dilutions of OVA at concentrations ranging from 0–10 ug/ml. Following a 72-h incubation, 1 μCi of [^3^H]thymidine (Amersham) was added to each well for 16 h. Using an automated cell harvester, the cells were collected onto glass microfiber filter, and the radioactive labeling incorporation was measured by a Wallac Betaplate liquid scintillation counter.

### Detection of CD8 T cell mediated caspase-3 activation

Flow cytometry was used to assess ability of CD8 cells to mediate induction of apoptosis pathway in target cells. Briefly, CD8 T cells were isolated from spleens of experimental and control mice on day 8 after immunization using the Murine CD8 Subset Column Kit (R&D Systems, Minneapolis, MN) according to the manufacturer's instructions. Cultures were prepared in RPMI 1640 medium (Invitrogen, CA) supplemented with 10% fetal bovine serum (FBS), 100 U penicillin G, 100 μg/ml of streptomycin sulfate and 2 mM L-glutamine. To test the CD8 effector function in terms of caspases-3 activating ability, the freshly isolated CD8 cells (effector cells, E) were mixed with target tumor cells (T) labeled with CellTracker Green dye at E:T ratios equal to 50:1, 25:1, 12.5:1, 6.25:1. As targets we used B16 melanoma cells, porcine splenocytes, murine splenocytes, as well as porcine trophoblasts and murine trophoblasts isolated by enzymatic dissociation with collagenase I and percoll density sedimentation as described [26,27]. After a 3 hour co-incubation, the effector:target mixtures were washed, fixed and permeabilized before staining with PE-labeled anti-Caspase-3 antibodies (BD Pharmingen, CA). After incubation (20 minutes, 4°C) and washing, the number of activated caspase-3 positive apoptotic cells was detected in CellTracker Green-positive target cells population and then the percentage of cells with activated caspases-3 were calculated using CellQuest software.

### ELISA

The supernatants from recall response T cell cultures were harvested at 24 hour incubation and assessed for IFN-γ and IL-4 by ELISA. Cytokine-specific ELISA (Endogen, Rockford, IL) was used for detecting cytokine concentrations in culture supernatants according to the manufacturer's instructions using a Benchmark Microplate Reader (Bio-Rad, Hercules, CA).

### B16 tumor model

For induction of tumor growth, 5 × 10^5 ^B16 American Type Culture Collection (Manassas, VA) cells were injected subcutaneously into the hind limb flank. Tumor growth was assessed every 3 days by two measurements of perpendiculardiameters by a caliper, and animals were sacrificed when tumors reached a size of 1 cm in any direction. Tumor volume was calculated by the following formula: (the shortestdiameter^2 ^× the longest diameter)/2

### Adoptive transfer

Groups of 8 mice immunized with either xenogeneic placental extract, saline, or control xenogeneic liver placental extract were challenged on day 7 with 5 × 10^5 ^B16 cells and observed for an additional 18 days. Subsequently, mice were sacrificed and CD4+, and CD8+ cells were isolated using the Murine CD4 Subset Column Kit, and Murine CD8 Subset Column Kit (R&D Systems), respectively. 10^7 ^CD4 and/or CD8 cells were transferred to naïve C57/BL6 mice intravenously at time of tumor challenge with 5 × 10^5 ^B16 cells.

### Statistical analysis

Data is presented in a primarily descriptive fashion. All experiments used 4–8 mice per treatment group and were repeated at least 3 times. For some experiments differences in tumor size were analyzed using the two-tailed Student's t test with significance determined at p < 0.05.

## Results

### Prophylaxis of tumor growth by Xenogeneic Placental Extract (XPE)

**Figure 1:**
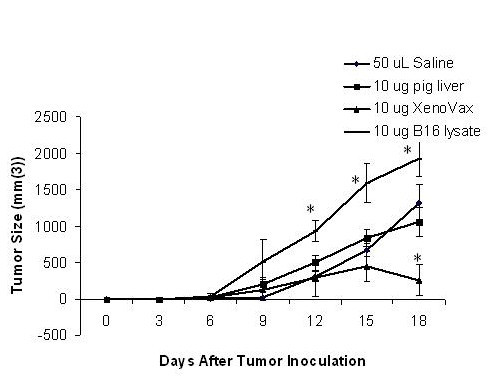
**Antitumor effects of XPE**. XPE preparations were dissolved into sterile, injection-grade PBS at a concentration of 2 mg/ml, and injections of 50 uL (total mass 10 ug) were performed subcutaneously into C57/BL6 mice 7 days before tumor challenge. 5 × 10^5 ^B16 murine melanoma cells were injected subcutaneously into the hind limb flank of female 6–8 week-old C57BL/6. Tumor growth was assessed every 3 days by two measurements of perpendicular diameters by a caliper, and animals were sacrificed when tumors reached a size of 1 cm in any direction. *p < 0.05, Student's T test compared to saline treated.

Induction of tumor immunity using xenogeneic antigens, and whole cells, has been previously reported [19-25]. However these studies have utilized a variety of immunization regimens, with multiple immunizations prior to tumor challenge. We sought to determine whether the potency of the anti-xenogeneic response to the xenogenic placental extract (XPE) would be sufficient to induce tumor immunity in a single dose injection. Accordingly, C57/BL6 mice were immunized subcutaneously with 10 μg of XPE, followed by a challenge with 5 × 10^5 ^B16 murine melanoma cells. In order to differentiate between the non-specific immune stimulatory activity of xenogenic tissue injection and XPE, we used as a control a preparation of xenogeneic hepatic cells prepared in a manner identical to XPE. Although B16 cells have been previously described as being poorly immunogenic, numerous investigators have used them for tumor vaccination [28,29], for this reason the other control group we have used consisted of B16 protein extracts purified using the method. As seen in Figure 1, in comparison to saline treated, mice immunized with the XPE had a significant reduction of tumor growth. The effect of xenogenic hepatic tissue immunization was similar to that of the saline in that tumor growth was not significantly altered. In contrast, growth of tumors in mice immunized with B16 extracts was actually accelerated in comparison to the saline or hepatic tissue extract immunized mice. Such an accelerated tumor growth may have been due to the positive influence of certain immune responses to tumor progression as seen by others using this model [30]. Overall, these data suggest that a single immunization with XPE is sufficient to induce a prophylactic antitumor response.

### XPE synergy with B16 vaccine

**Figure 2:**
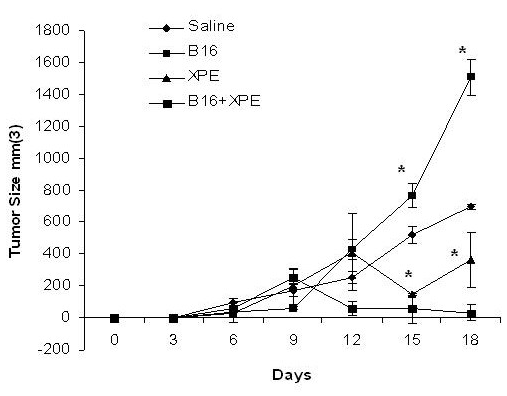
**XPE augments immunity of B16 lysate "vaccine"**. XPE and B16 melanoma cell lysates were prepared as described in Figure 1 using freeze-thaw cycles. C57/BL6 mice were immunized with XPE alone or co-mixed with the B16 melanoma lysate diluted in PBS at a concentration of 2 mg/ml, and injections of 50 uL (total mass 10 ug). Tumor growth was assessed every 3 days by two measurements of perpendicular diameters by a caliper, and animals were sacrificed when tumors reached a size of 1 cm in any direction. *p < 0.05, Student's T test compared to saline treated.

The demonstration of enhanced tumor growth in mice immunized with B16 melanoma extracts suggested to us that an immune response was induced, however the response was not therapeutically desired since it actually enhanced tumor growth. We therefore sought to determine whether XPE would be capable of modifying the B16-induced response in order to cause inhibition of tumor growth instead of augmentation. The rationale being that XPE not only contains tumor antigens, but also may be capable of triggering activation of various innate effector cells such as dendritic cells [31]. In order to use conditions similarly to the clinic, in this set of experiments mice were immunized at the same time as tumor inoculation. In agreement to the previously described experiments, immunization with B16 extracts lead to an enhancement of tumor growth, while immunization with XPE led to inhibition (Figure 2). Interestingly, the co-administration of B16 extracts and XPE led to a synergistic inhibition of tumor growth (Figure 2). This ability to potentiate immune response to B16 cells seemed to be tumor-specific in that when EL-4 cell extracts were used for co-immunization, no synergy of inhibition was observed (data not shown). In summary, these experiments demonstrate that XPE is not only capable of inducing antitumor immunity, but also can synergize with sources of antigen at inducing such immunity.

### Dendritic cell immunization

**Figure 3:**
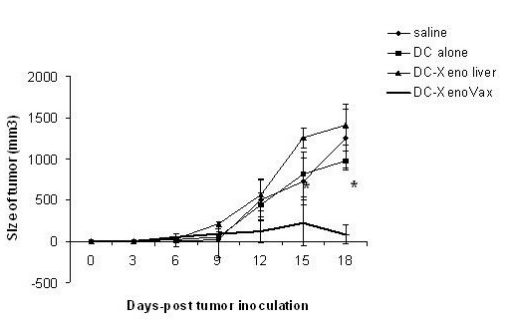
**Induction of anti-cancer immunity by XPE-pulsed DC**. Day 7 bone marrow-derived DC were pulsed with 10 μg/ml XPE for 24 h and injected s.c. (5 × 10^5 ^cells/mouse) into syngeneic C57BL/6 mice. A concurrent injection of 5 × 10^5 ^B16 melanoma cells was administered. Tumor growth was assessed every 3 days by two measurements of perpendicular diameters by a caliper, and animals were sacrificed when tumors reached a size of 1 cm in any direction. *p < 0.05, Student's T test compared to saline treated.

**Figure 4:**
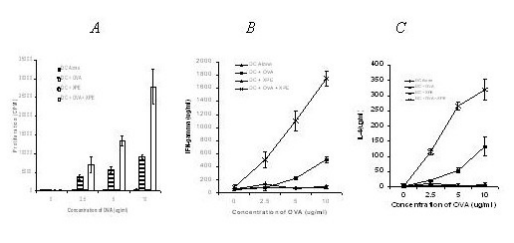
**Potentiation of immunity by XPE pulsed DC**. C57/BL6 mice were injected were injected subcutaneously with 5 × 10^5 ^bone marrow-derived DC pulsed with either 10 μg/ml XPE, and/or 10 μg/ml OVA for 24 hr. Mice were sacrificed 14 days post inoculation and splenocytes were cultured with increasing concentrations of OVA in vitro. Cultures were assessed for: *A*) Proliferation; *B*) IFN-γ production; and *C*) IL-4 production.

Previous experiments have demonstrated induction of clinically significant responses by immunizing patients with autologous dendritic cells (DC) pulsed with xeno-antigens [32]. Accordingly, we sought to determine whether XPE could be used as a source of antigen for pulsing DC. Murine BM-derived DC were generated according to standard protocols used by us [33], pulsed with XPE or porcine liver extracts on day 7 for 24 hours, and injected subcutanously on the same day as tumor challenge. DC were confirmed to be >95% purity as detected by CD11c expression (data not shown). Similarly to experiments using direct immunization, XPE pulsed DC induced a potent suppression of tumor growth, whereas DC pulsed with control xenogenic liver extract did not evoke tumor protection (Figure 3). In order to determine whether the XPE had an adjuvant effect on DC induction of immunity similar to the synergy observed between XPE and B16 extracts in the previous experiments, we co-pulsed DC with the antigen OVA alone, with XPE alone, or the combination in non-tumor bearing C57/BL6 mice. T cell recall responses were assayed on day 14 after immunization. As seen in Figure 4, a stronger induction of proliferation (*4a*), IFN-γ (*4b*) and IL-4 (*4c*) was observed in recall response to OVA when DC were co-cultured with XPE before injection as opposed to non-coculture. Overall these data suggest that XPE can act both as a source of antigen for immune response induction by administration through DC, and can also act as an adjuvant to DC antigen presentation.

### XPE-induced protection is CD4+ T cell dependent

**Figure 5:**
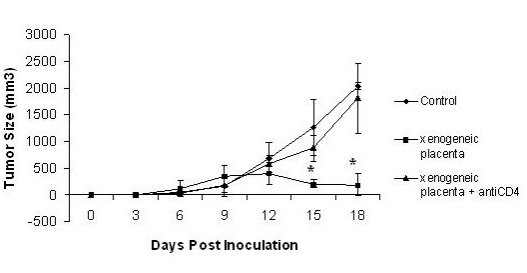
**XPE-induced anti-tumor immunity is CD4 dependent**. CD4 cell depletion was accomplished in C57/BL6 mice by intravenous injection of anti-CD4 monoclonal antibody (clone GK1.5) on day -5, day -3, day 0, day 1, day 3, and day 5 at a concentration of 150 ug/mouse. XPE was injected on day 0, which was also the timepoint of tumor injection (5 × 10^5 ^B16) Tumor growth was assessed every 3 days by two measurements of perpendicular diameters by a caliper, and animals were sacrificed when tumors reached a size of 1 cm in any direction. *p < 0.05, Student's T test compared to saline treated.

Of possible mechanisms utilized by XPE to cause protection from B16 melanoma, we hypothesized that the adaptive immune response may be involved. Accordingly, we depleted CD4 T cells by intravenous injection of anti-CD4 monoclonal antibody (clone GK1.5) on day -5, day -3, day 0, day 1, day 3, and day 5 at a concentration of 150 ug/mouse. Depletion (>95% compared to control Ig treated) was confirmed by flow cytometry (data not shown). As seen in Figure 5, immunization with XPE did not induce protection in the CD4 depleted mice, but induced protection in mice having a wild-type CD4 immune response. These data suggest the importance of adaptive immunity, as coordinated by CD4 T cells, at mediating the cancer inhibitory activities of XPE.

### XPE-induced anti-tumor response is associated with generation of CD8 cells capable of activating caspase-3

**Figure 6:**
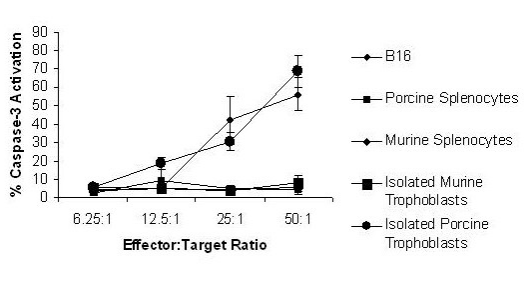
**XPE-induction of caspase-3 activating CD8 cells**. CD8 T cells were isolated from spleens of experimental and control mice on day 8 after immunization with XPE and mixed at the indicated ratios with the indicated target cells. Caspase-3 activation was quantified as percentage of target cells positive for anti-activated caspase-3 antibody staining by flow cytometry. The results are representative of 3 independently performed experiments.

In order to determine whether XPE induced anti-tumor immunity is associated with induction of cytotoxic T cells, C57/BL6 mice were immunized with 10 μg of XPE, allogeneic placental extract (APE), or xenogenic liver extract and sacrificed on day 14. CD8 T cells were extracted from splenocytes and incubated with labeled target cells and subsequently treated with anti-activated caspase-3 antibody. Assessment by flow cytometry for caspase-3 activation on target cells indicated a dose-dependent induction of caspase 3 in B16 melanoma cells, as well as porcine trophoblasts, but not in murine trophoblasts, porcine splenocytes, or murine splenocytes (Figure 6). When effector cells were isolated from mice immunized with APE or xenogenic liver extracts, no activation of caspase-3 was detected regardless of effector:target ratio (data not shown). These results support the notion that CD8 T cells are generated subsequent to XPE administration that are capable of inducing the activation of apoptotic pathways in target cells. The observation that neither APE immunization, nor xenogeneic liver extract immunization lead to activation of CD8 cells with caspase-3 inducing activity suggests a somewhat specific effect of xenogenicity on induction of anti-tumor effects of XPE, as well as that the effect is not mediated by non-placental tissue.

### Transfer of XPE-induced protection requires CD4 and CD8 T cells

**Figure 7:**
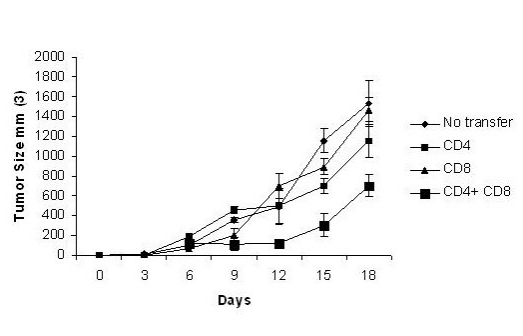
**Adoptive transfer of XPE-induced immunity**. Mice were immunized with XPE, challenged on day 7 with 5 × 10^5 ^B16 cells, observed for an additional 18 days after which CD4+, and CD8+ cells were harvested from splenocytes. CD4 and CD8 cells were transferred alone or together at a concentration of 10^7 ^cells/mouse to naïve C57/BL6 mice intravenously at time of tumor challenge with 5 × 10^5 ^B16 cells.

To conclusively demonstrate that the induction of lymphoid effector cells subsequent to XPE vaccination is causative of tumor regression, we performed a standard adoptive transfer experiment. Groups of 8 mice were immunized with XPE or xenogeneic liver extracts as described in Figure 1, challenged with tumors on day 7, and subsequently sacrificed 18 days subsequent to tumor challenge. CD4 and CD8 cells were isolated and transferred into naïve C57/BL6 mice at the time of tumor challenge. As seen in Figure 7, only the co-transfer of both CD4+ and CD8+ cells was capable of inducing tumor inhibition. Adoptive transfer of immune cells from mice immunized with xenogenic liver extracts had no inhibitory effect (data not shown).

## Discussion

It has been reported that immunization with placental extracts possesses clinical efficacy toward a variety of tumor types clinically, as previously reviewed by Harandi [34]. Unfortunately, randomized clinical trials have not been performed and the data is at best anecdotal. Conceptually, vaccination with placental extracts would be capable of eliciting immunological responses not only to marker antigens shared between the placenta and neoplasia such as hCG, MAGE, BAGE [17,18], but also to functional proteins that tumors require to maintain malignancy. For example, stimulation of immunity against MMPs found on placental tissue may theoretically cause inhibition of tumor MMP activity and reduction in tissue invasiveness and metastasis. Indeed, while others have previously demonstrated inhibition of tumor growth after immunization with MMP-2 [22], it was recently observed that human melanoma cells uptake MMP-2 in an alpha v beta3-dependent manner, and present peptides thereof to HLA*0201-restricted T cells [35].

In addition to MMPs, placental formation in normal pregnancy is dependent on two other main functional activities, the first being suppression of immune responses in a selective or semi-selective manner, and the second being ability to rapidly induce angiogenesis to form the appropriate interface for maternal-fetal nutrient transmission. It is established that tumors also require induction of immune suppression, as well as angiogenesis for their survival, and indeed the higher immune suppression and angiogenesis found in tumors, the worse the patient prognosis is. The sharing between placental cells and cancer cells of immune suppression/immune evasion molecules such as fas ligand (Fas-L) [7], DAF [8], CD55 [9], IL-10 [10], MICA [36], HLA-G and indolamine 2,3 dioxygenase [37], as well as molecules of angiogenesis such as VEGF [11], placental growth factor [38], angiopoietin [39], FGF, EGF, and TGF-beta [40] suggests not only a functional, but also a molecular homology between placenta and cancer cells.

The knowledge that placental extracts may be stimulatory of anticancer immune responses has been postulated to account for the lower incidence of certain tumors in multi-parous women [41]. Indeed older studies using placental immunization in mouse models have demonstrated some efficacy in tumor inhibition as reviewed by Harandi [34]. Our approach to utilization of placental extracts was mindful of the Immunostimulatory Hypothesis proposed by Prehn [42], suggesting that the immune response may in some cases actually contribute to tumor growth. With this in mind, we sought to specifically use a potent means of inducing a "danger signal" while concurrently providing a multivalent tumor antigenic source. Based on the hyperacute nature of xenogenic rejection [43], the multitude of data supporting superior breaking of tolerance using xenogenic variants of self-proteins [44-46], and the fact that autoimmune disease is evoked only by xenogeneic self antigens in animal models of autoimmune disease [47,48], we decided to immunize C57/BL6 mice with xenogenic placental extracts (XPE) and observe antitumor effects.

We have demonstrated that immunization with a preparation of xenogenic placental extract induced a CD4-dependent immune response capable of inhibiting growth of B16 melanoma cells in the C57/BL6 mouse. Demonstration of CD8 activity in the induction of tumor regression was performed in experiments showing that freshly isolated CD8 cells from immunized mice were capable of inducing both tumor cell, as well as xenogeneic trophoblast apoptosis cascades. Immune-mediated clearance seemed to be specific to the XPE since immunization with control porcine liver extracts did not mediate protection. Additionally, the xenogeneic component of XPE seems to be critical since apoptosis-inducing cells were not detected in mice immunized with allogeneic placental extracts. Although we did not elucidate specific antigenic targets of xenoplacental immunization, preliminary data seems to suggest that inhibition of neoangiogenesis may be one of the potent mechanisms. Studies using purified immunoglobulin from XPE immunized mice demonstrate the ability to inhibit in vitro proliferation of endothelial cells. This work is still in progress and will be reported in a subsequent publication.

The ability of XPE to inhibit tumor growth may not be surprising given the high concentration of endothelial cells in placental tissue, and previous reports that immunization with xeno-endothelial cells causes anticancer responses [25,49]. However, the potent ability of XPE to act as a Th1 and Th2 adjuvant for HEL-specific recall responses is surprising. Indeed it has been speculated that xenogenic antigens may intrinsically be stimulatory of dendritic cell function, however to our knowledge, this is the first report actually demonstrating this. Future experiments will assess the effect of XPE on dendritic cell maturation, and function in vitro in order to identify whether the XPE was actually giving a direct signal to dendritic cells, or whether the augmentation of DC activation occurs after the re-introduction of pulsed DC in vivo. Nevertheless, it appears that XPE has a potent immune enhancing property since it was able to potentiate the anti-B16 response of the B16 lysate vaccine in addition to the anti-HEL response.

Although numerous tumor antigens are currently under intense investigation, the ability for a multi-epitopic composition such as XPE to induce immunity that is transferable to naïve recipients is somewhat surprising given the lack of immunological adjuvants or multi-injection regimens used in other studies. In conclusion, XPE appears to be a potent stimulator of anticancer immune responses in a CD4 and CD8 T cell dependent manner. The characterization of both cross-reactive tumor antigens, as well as the apparent ability to act as an immunological adjuvant, will provide interesting new avenues of research, with the possibility of developing novel, clinically applicable therapeutics from a relatively benign antigenic source.
